# Long-range axonal projections of transplanted mouse embryonic stem cell-derived hypothalamic neurons into adult mouse brain

**DOI:** 10.1371/journal.pone.0276694

**Published:** 2022-11-10

**Authors:** Miho Kawata, Yu Kodani, Mahito Ohkuma, Ei-ichi Miyachi, Yoko S. Kaneko, Akira Nakashima, Hidetaka Suga, Toshiki Kameyama, Kanako Saito, Hiroshi Nagasaki

**Affiliations:** 1 Department of Physiology I, Fujita Health University School of Medicine, Toyoake, Aichi, Japan; 2 Department of Physiology II, Fujita Health University School of Medicine, Toyoake, Aichi, Japan; 3 Department of Health and Nutrition, Faculty of Health and Science, Nagoya Women’s University, Nagoya, Aichi, Japan; 4 Biochemistry and Molecular Cell Biology, Faculty of Pharmacy, Gifu University of Medical Science, Kani, Gifu, Japan; 5 Department of Physiological Chemistry, Fujita Health University School of Medicine, Toyoake, Aichi, Japan; 6 Department of Endocrinology and Diabetes, Nagoya University Graduate School of Medicine, Nagoya, Aichi, Japan; University Hospital Wurzburg, GERMANY

## Abstract

The hypothalamus is comprised of heterogenous cell populations and includes highly complex neural circuits that regulate the autonomic nerve system. Its dysfunction therefore results in severe endocrine disorders. Although recent experiments have been conducted for *in vitro* organogenesis of hypothalamic neurons from embryonic stem (ES) or induced pluripotent stem (iPS) cells, whether these stem cell-derived hypothalamic neurons can be useful for regenerative medicine remains unclear. We therefore performed orthotopic transplantation of mouse ES cell (mESC)-derived hypothalamic neurons into adult mouse brains. We generated electrophysiologically functional hypothalamic neurons from mESCs and transplanted them into the supraoptic nucleus of mice. Grafts extended their axons along hypothalamic nerve bundles in host brain, and some of them even projected into the posterior pituitary (PPit), which consists of distal axons of the magnocellular neurons located in hypothalamic supraoptic and paraventricular nuclei. The axonal projections to the PPit were not observed when the mESC-derived hypothalamic neurons were ectopically transplanted into the substantia nigra reticular part. These findings suggest that our stem cell-based orthotopic transplantation approach might contribute to the establishment of regenerative medicine for hypothalamic and pituitary disorders.

## Introduction

The hypothalamus is a highly complex region of the brain. This complexity arises from the heterogenous nuclei comprising a wide variety of neurons, such as arginine vasopressin (AVP), oxytocin (OXT), melanin-concentrating hormone (MCH), orexin, proopiomelanocortin (POMC), agouti-related peptide (AgRP) and neuropeptide Y (NPY). These neurons project their axons across the whole brain and form elaborate neural networks that manage diverse physiological functions, such as feeding, water balance, thermoregulation, wake-sleep cycles, stress and social and sexual behaviors [1–4].

Among the hypothalamic neurons, the AVP and OXT neurons send their axons into the posterior pituitary (PPit) and release peptide hormones. A deficiency of AVP causes central diabetes insipidus (CDI), which is characterized by polyuria and polydipsia. CDI is usually a benign disease, but it can be life-threatening if patients have osmoreceptor dysfunction, hypernatremia and dehydration [5]. Thus, the establishment of a definitive treatment of CDI is eagerly awaited.

Stem cell therapy is a promising tool for treating not only CDI but also other hypothalamic disorders. Several groups have reported the differentiation of hypothalamic neurons from mouse embryonic stem cells (mESCs) [6] or pituitary cells from mESCs [7] or human pluripotent stem cells (hPSCs) [8, 9]. A recent study successfully generated hypothalamus-pituitary organoids from human induced pluripotent stem cells (hiPSCs), in which adrenocorticotropic hormone (ACTH) and corticotropin-releasing hormone (CRH) cells coexisted, responding to the external low-glucose environment and increasing ACTH secretion through their own CRH-ACTH pathway [10]. In addition, several studies have demonstrated that ectopically transplanted *in vitro*-generated hypothalamic neurons and pituitary cells function properly *in vivo*. For example, hPSC-derived hypothalamic neurons transplanted into a newborn mouse brain were able to survive and maintain their hypothalamic identity for at least 18 months after transplantation [11], and kidney subcapsular or subcutaneous transplantation of hPSC-derived pituitary cells increased the hormone levels and rescued the physical activity and survival of hypopituitary model animals [12, 13]. However, to our knowledge, no studies have investigated the orthotopic transplantation of stem cell-derived hypothalamic neurons or pituitary cells.

The present study therefore investigated whether mESC-derived hypothalamic neurons could survive and form neural networks when orthotopically transplanted into the supraoptic nucleus (SON) of an adult host brain.

## Materials and methods

### mESC culture

All experiments using mESCs were approved by the Ethics Committee of Fujita Health University (protocol number AP16029-MD2). mESCs (EB5, RCB#AES0151, RRID: CVCL_J648) were maintained on 0.1% gelatin (Sigma-Aldrich, Saint-Louis, MO, USA; Cat# G2500, CAS# 9000-70-8) -coated plates in the maintenance medium composed of G-MEM (FUJIFILM, Wako Pure Chemical Corporation, Osaka, Japan; Cat# 078–05525) supplemented with 1% fetal bovine serum (FBS) (CCB, NICHIREI BIOSCIENCE, Tokyo, Japan; Cat# 171012), 10% KnockOut serum replacement (KSR) (Gibco; Thermo Fisher Scientific, Inc., Waltham, MA, USA; Cat#10828028), 0.1 mM non-essential amino acids (MEM NEAA) (Gibco; Cat# 11140050), 1 mM pyruvate (Sigma-Aldrich; Cat# S8636, CAS# 113-24-6), 0.1 mM 2-mercaptoethanol (Kanto Chemical Co., Inc., Tokyo, Japan; Cat# 25099–30, CAS# 60-24-2), Blastcidin (20 μg/ml; Wako; Cat# 029–18701, CAS# 3513-03-9) and 2,000 U/ml LIF (Wako; Cat# 195–16053) at 37°C in 5% CO_2_.

### Induction of hypothalamic precursor cells

Each cell was cultured according to the SFEBq protocol [6]. In brief, mESCs were enzymatically dissociated to single cells in 0.25% Trypsin-EDTA (Wako; Cat# 201–16945) and quickly reaggregated in differentiation medium (3,000 cells per well) using 96-well U-bottom low cell-adhesion plates (Sumitomo Bakelite Co., Ltd., Tokyo, Japan; Cat# MS-9096U). The differentiation medium was growth factor-free CDM (gfCDM), which contains Iscove’s modified Dulbecco’s medium (Gibco; Cat# 31980030) /Ham’s F-12 (Gibco; Cat# 31765035) 1:1, 1×chemically defined lipid concentrate (Gibco; Cat# 11905031), monothioglycerol (450 μM; Sigma-Aldrich; Cat# M6145, CAS# 96-27-5) and 5 mg/ml purified bovine serum albumin (BSA, Sigma-Aldrich; Cat# A9418, CAS# 9048-46-8). The differentiation medium was used from days 0–7. To regulate Sonic hedgehog (Shh) signal transduction, 10 nM SAG (Cayman Chemicals, Ann Arbor, MI, USA; Cat# 11914, CAS# 912545-86-9) was added to the culture from day 4 for induction of ventral hypothalamic neurons, such as POMC, AgRP and NPY.

### Neuronal differentiation

For neuronal differentiation, on day 7, the medium was changed to the DFK medium (D-MEM/Ham’s F12 without HEPES [Wako; Cat# 048–29785] supplemented with 35 mM glucose [Wako; Cat# 079–05511, CAS# 50-99-7], 10% KSR, 1×penicillin/streptomycin [Wako; Cat# 168–23191]). On day 10, half medium was changed to the neuronal differentiation medium (NDM) (D-MEM/Ham’s F12 [Wako; Cat# 042–30555] supplemented with 35 mM glucose [Wako], 1×penicillin/streptomycin [Wako], 1×N2 supplement [Wako; Cat# 141–08941], 2% MACS NeuroBrew-21 without vitamin A [Miltenyi Biotec, Auburn, CA, USA; Cat# 130-097-263] and 10 ng/ml CNTF [Wako; Cat# 032–18851]). On day 14, cells were dissociated from aggregates by neuron dissociation solution (Wako; Cat# 297–78101) containing 10 μM Y-27632 (Wako; Cat# 034–24024, CAS# 331752-47-7) for 30 min at 37°C. After removal of the enzyme solution by centrifugation, the collected cells were plated onto Matrigel (CORNING, Corning, NY, USA; Cat# 354230)/laminin (Corning; Cat# 354232)/polyD-lysine (Corning; Cat# 354210)-coated 6-well plates (Corning; Cat# 353046) at a density of 400,000 cells per well in NDM medium with the addition of 10% FBS, 50 ng/ml BDNF (BioLegend, San Diego, CA, USA; Cat# 788904) and 50 ng/ml NT3 (BioLegend; Cat# 598202). For the dissociation culture of Shh-treated cells, cells were plated in NDM medium without CNTF, and 50 ng/ml BDNF and 10 nM SAG were added.

### *In vitro* AAV transduction

After 28 days of SFEBq culture, differentiated cells were incubated with AAV-containing medium composed of AAV-CAG-tdTomato (#59462-AAV1; 1.9×10^13^ GC/ml; Addgene, Watertown, MA, USA), D-MEM/Ham’s F12 supplemented with 35 mM glucose, and 1×penicillin/streptomycin for 2 h at 37°C followed by the addition of fresh NDM medium along with 10% FBS, 50 ng/ml BDNF and 50 ng/ml NT3. The next day, a full medium change was performed. At least 5 days after transduction, cells were used for transplantation.

### Generation of knock-in ES cell lines

To knock-in EGFP at the mouse AVP gene, Xanthomonas TAL Nucleases (XTN; Transposagen Gene Editing Kit; Transposagen Biopharmaceuticals, Inc., Lexington, KY, USA) were designed to cause a mutation at the first exon of the vasopressin precursor gene around the initial ATG. For recombination, homology arms (5’: 0.9 kbp, 3’: 0.9 kbp) were amplified by polymerase chain reaction (PCR) from 129J mouse genomic DNA (from EB5 ES cells). The cDNA of enhanced GFP (EGFP; BD Biosciences) was fused into the first exon of the AVP. A PGK promoter-driven hygromycin-resistance selection cassette flanked by loxP sites was inserted downstream of EGFP. Vectors for the XTN pair with linearized targeting vector were co-transfected by electroporation (Mouse ES Cell Nucleofector™ kit and Nucleofector II; Lonza, Basel, Switzerland; Cat# VPH-1001) into EB5 ES cells. Homologous recombinant ES cells were selected with hygromycin and then screened by PCR. Targeted clones were confirmed by a PCR analysis with the 3’, 5’ and neo probes. The floxed PGK-hygro cassette was removed from subclones by transient transfection with Cre-expressing plasmid (pCAG-Cre:GFP; Addgene, #13776) using Lipofectamine 2000 (Invitrogen; Thermo Fisher Scientific, Inc. Cat# 11668030). The resultant subclones exhibited abilities to differentiate into hypothalamic neurons, including vasopressin.

### Electrophysiology

For patch-clamp recording, the recording chamber dish was mounted on the stage of an upright microscope (BX-50; Olympus, Tokyo, Japan, RRID:SCR_018838). The indifferent electrode was an Ag-AgCl wire connected to the recording dish. Membrane voltages and currents were recorded in the whole-cell configuration using a patch-clamp amplifier (Axopatch 200B; Molecular Devices, San Jose, CA, USA, RRID:SCR_018866) linked to a computer [14–16]. The voltage-clamp and current-clamp procedures were controlled by the pCLAMP software (Molecular Devices, RRID:SCR_011323). Data were low-pass-filtered with a cut-off frequency of 5 kHz and then digitized at 10 kHz by an analog-to-digital interface (Digidata 1322A; Molecular Devices, RRID:SCR_021041). Culture cells were perfused at 1 ml/min with ringer solution bubbled with 100% O_2_. The composition of HEPES-buffered ringer solution (in mM) was 135 NaCl, 5 KCl, 2 CaCl_2_, 1 MgCl_2_, 10 glucose and 10 HEPES (pH adjusted to 7.4 with KOH). The recording pipette was filled with the following pseudo-intracellular solution (in mM): 140 KCl, 1 CaCl_2_, 2 MgCl_2_, 5 BAPTA and 10 HEPES (pH adjusted to 7.4 with KOH). The pipette resistance was 6–8 MΩ. The fluorescent dye Lucifer yellow (0.025%) was also added to the recording pipette to identify the shape of the recorded cells. Glutamate (Glu; 1 mM, 50 μl) or gamma-aminobutyric acid (GABA; 1 mM, 60 μl) was added to the perfused ringer solution in the recording chamber (1 ml volume). The final concentration of each transmitter did not exceed 50 μM (Glu) or 60 μM (GABA). Tetrodotoxin (1 μM TTX, a voltage-gated sodium channel blocker), or picrotoxin (10 μM PTX, an antagonist of GABA_A_ receptor) was applied through the bath. Electrophysiological experiments were performed at room temperature (RT; 23–25°C).

### Cell preparation for transplantation

Cells were pre-treated with 10 μM Y-27632 at least 1 h prior to dissociation. Cells were washed twice with phosphate-buffered saline (PBS) and enzymatically dissociated with neuron dissociation solution containing 10 μM Y-27632 for 10 min at 37°C. After removal of the enzyme solution by centrifugation, cells were resuspended at a density of 1 × 10^5^ to 8 × 10^5^ cells per mouse in medium composed of D-MEM/Ham’s F12 without HEPES supplemented with 35 mM glucose, 1×penicillin/streptomycin, 10% FBS and 10 μM Y-27632. Cells were kept on ice until transplantation.

### Transplantation of mESC*-*derived hypothalamic neurons

All animal experiments were approved by the Ethics Committee of Fujita Health University (protocol number AP16029-MD2). NOD/ShiJic-scidJcI (NOD/SCID) mice (Clea, Tokyo, Japan) were kept under a 12 h light/dark cycle with access to food and water *ad libitum*. Adult NOD/SCID mice (6 to 14 weeks old) were anesthetized with isoflurane and placed in a stereotaxic apparatus (Narishige, Tokyo, Japan; Cat# SR-6N). mESC-derived hypothalamic neurons were transplanted into the SON (n = 9) (from bregma: A -1.0 mm, L -1.0 mm, V +6.6 mm) or substantia nigra reticular part (SNr) (n = 8) (from bregma: A -3.0 mm, L -1.5 mm, V +4.6 mm) using a 22-gauge Hamilton needle (Hamilton Company, Reno, NV, USA; Cat# 90134) and microsyringe (Hamilton Company; Cat# 81001).

### Immunocytochemistry

Cells were seeded onto a Matrigel/laminin/polyD-lysine-coated cover glass (Matsunami Glass, Osaka, Japan; Cat# C013001) in culture plates. Cells were washed with PBS and fixed in 4% paraformaldehyde (PFA) for 0.5–1 h at RT. After fixation, cells were blocked with blocking buffer containing 5% normal donkey serum (Sigma-Aldrich; Cat# D9663), 0.1% Triton X-100 and 0.02% NaN_3_ in PBS for 1 h at RT. Cells were then incubated overnight at 4°C in blocking buffer with primary antibodies. After washing 3 times in PBS, cells were incubated 1 h at RT in blocking buffer with secondary antibodies. The antibodies used in this study are listed in S1 Table. Nuclear staining was performed with DAPI (Dojindo Laboratories, Kumamoto, Japan; Cat# D523, CAS# 28718-90-3). After washing three times in PBS, cell-seeded cover glasses were mounted onto glass slides using Fluoromount (DBS Diagnostic Biosystems, Pleasanton, CA, USA; Cat# K024). Imaging data were acquired using an inverted fluorescence microscope (DMI6000B; Leica Microsystems, Manheim, Germany, RRID:SCR_020216) and analyzed with the Fiji software (RRID:SCR_002285).

### Immunohistochemistry

To alleviate suffering, mice were deeply anesthetized with isoflurane before perfusion fixation. Mice were transcardially perfused with PBS followed by 4% PFA, and then their brains were collected. The brains were postfixed overnight in 4% PFA and cryoprotected in sucrose solutions of increasing concentrations (10%, 20% and 30%) overnight at 4°C. Brains and pituitaries were coronally sectioned (10, 30 or 50 μm thickness) on a freezing microtome (CM1950; Leica Microsystems, RRID:SCR_018061) and mounted onto CREST-coated slides (Matsunami Glass; Cat# SCRE). Each slice was placed in PBS and washed 3 times followed by 1 h incubation with blocking buffer. Slices were then incubated overnight at 4°C in blocking buffer with primary antibodies. After washing 3 times in PBS, slices were incubated for 1 h at RT in blocking buffer with secondary antibodies. The antibodies used in this study are listed in S1 Table. Slices were then washed three times in PBS and coverslipped with Fluoromount. Imaging data were acquired using a DMI6000B or BIOREVO BZ-9000 (Keyence, Osaka, Japan, RRID:SCR_015486) fluorescence microscope or an LSM 710 confocal microscope (Carl Zeiss Microscopy, Jena, Germany, RRID:SCR_018063) and analyzed with the Fiji software. For the observation of grafts and axons, Z stack slices were obtained using an LSM 710 with a 40× objective in the brain (10 stacks at 1.17 μm intervals) or with a 63× objective in the PPit (12–15 stacks at 1.33 μm intervals). Z stack slices were displayed as maximum intensity projection images.

### Imaging and data analysis of the axonal projection of transplanted mESC-derived hypothalamic neurons

To examine into which brain regions the axons from the grafts projected, imaging data were acquired using an inverted fluorescence microscope (DMI6000B; Leica). Each brain region was determined with reference to the mouse brain atlas [17], (www.brain-map.org; [18]). The number and percentage of mice in which tdTomato fibers were observed in each brain region were calculated. The density of tdTomato-positive fibers was estimated by calculating the mean percentage of tdTomato-positive area (μm^2^) in each brain region using Fiji. A “Yen” threshold was applied, and then the “analyze particles” function was used to calculate tdTomato-positive areas (μm^2^) in each brain regions. By assigning 100% to the total tdTomato-positive areas of the SON- or SNr-grafted group, the mean percentage of tdTomato-positive areas in each brain region was calculated. A mean percentage of 21%-30% was considered high (+++), 11%-20% was considered medium (++), >0%-10% was considered low (+), and 0% was considered (-).

### Cell quantification and statistical analysis

Surviving cell numbers were estimated by considering the graft volume and numerical density. Every third section was used for quantification. The graft volume was quantified according to the protocols described previously [19, 20]. The following formula was used: *V* = (Σ*A*×*M*)/*f*, where *V* = volume (μm^3^), *A* = Area of the graft (μm^2^), *M* = section thickness (μm) and *f* = section frequency. The areas of the graft were captured using a DMI6000B with a 10× objective and measured with the Fiji software program.

To quantify the numerical density, Z-stack images were captured using an LSM 980 confocal microscope (Carl Zeiss Microscopy) with a 40× objective (40 stacks at 0.35 μm intervals), and tdTomato/DAPI double-positive cells were counted using the Imaris software program (OXFORD Instruments, Zurich, Switzerland; RRID:SCR_007370). The crude total cell number was calculated according to the protocols described previously [21]. The following formula was used: *C* = *Nv*×*V*, where *C* = crude total cell number, *Nv* = numerical density of tdTomato/DAPI double-positive cells and *V* = graft volume (μm^3^). The crude total cell number was corrected by the Abercrombie method [22]. Abercrombie’s formula is as follows: *P* = *C*×(*M*/*L*+*M*). where *P* = true count cell number, *C* = crude total cell number, *M* = section thickness (μm) and *L* = the average length of the nuclei (μm). The graft survival rate was calculated as follows: *R* = (*P*/grafted cell numbers) × 100, where *R* = the graft survival rate and *P* = true count cell number. The results were presented as the mean ± standard error of the mean (SEM). An unpaired Student’s *t*-test was used for comparisons between the SON- and SNr-grafted groups. A p-value <0.05 was considered statistically significant. The minimal data set for the calculation of the graft survival rate is shown in S1 Data.

## Results

### Generation of hypothalamic neurons from mESCs

**Fig 1:**
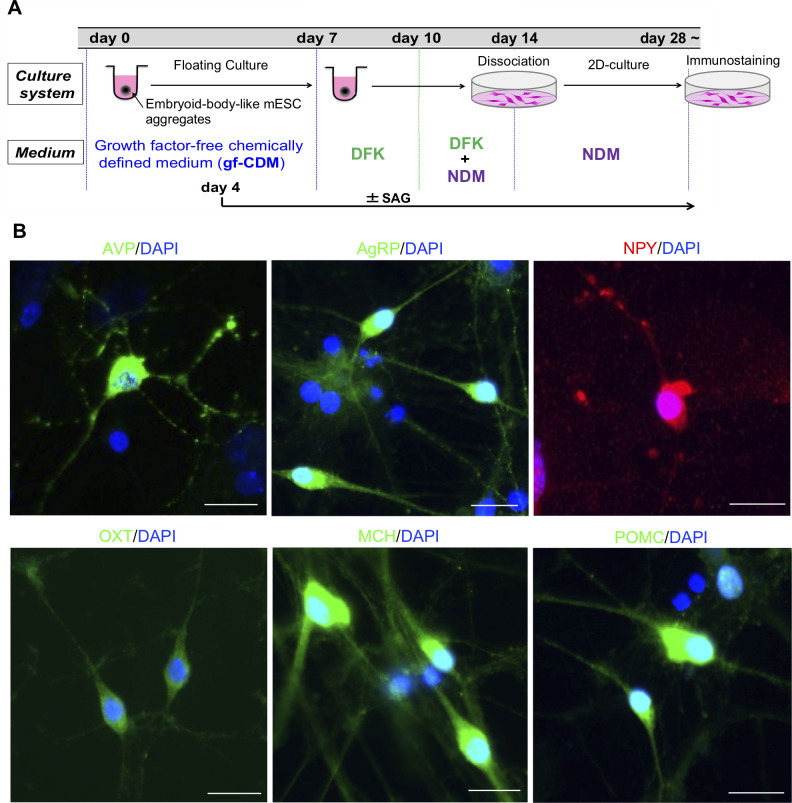
Generation of various hypothalamic neurons from mESCs. (A) Schematic illustration of hypothalamic differentiation from mESCs. (B) Immunostaining of dissociation culture for various hypothalamic neuronal markers (AVP, AgRP, NPY, OXT, MCH and POMC). Scale bars: 20 μm.

To generate hypothalamic neurons from mESCs, we used the SFEBq/gfCDM method [6] with some modifications (Fig 1A). On day 14, aggregates were transferred to two-dimensional culture and then matured into hypothalamic neurons. Consistent with a previous study, we observed cells expressing AVP, AgRP, NPY, OXT, MCH and POMC in the culture at 28 days after SFEBq (Fig 1B). These results suggest that our modified culture protocol induced appropriate differentiation of hypothalamic neurons from mESCs.

### Electro-physiological properties of mESC-derived hypothalamic neurons

**Fig 2:**
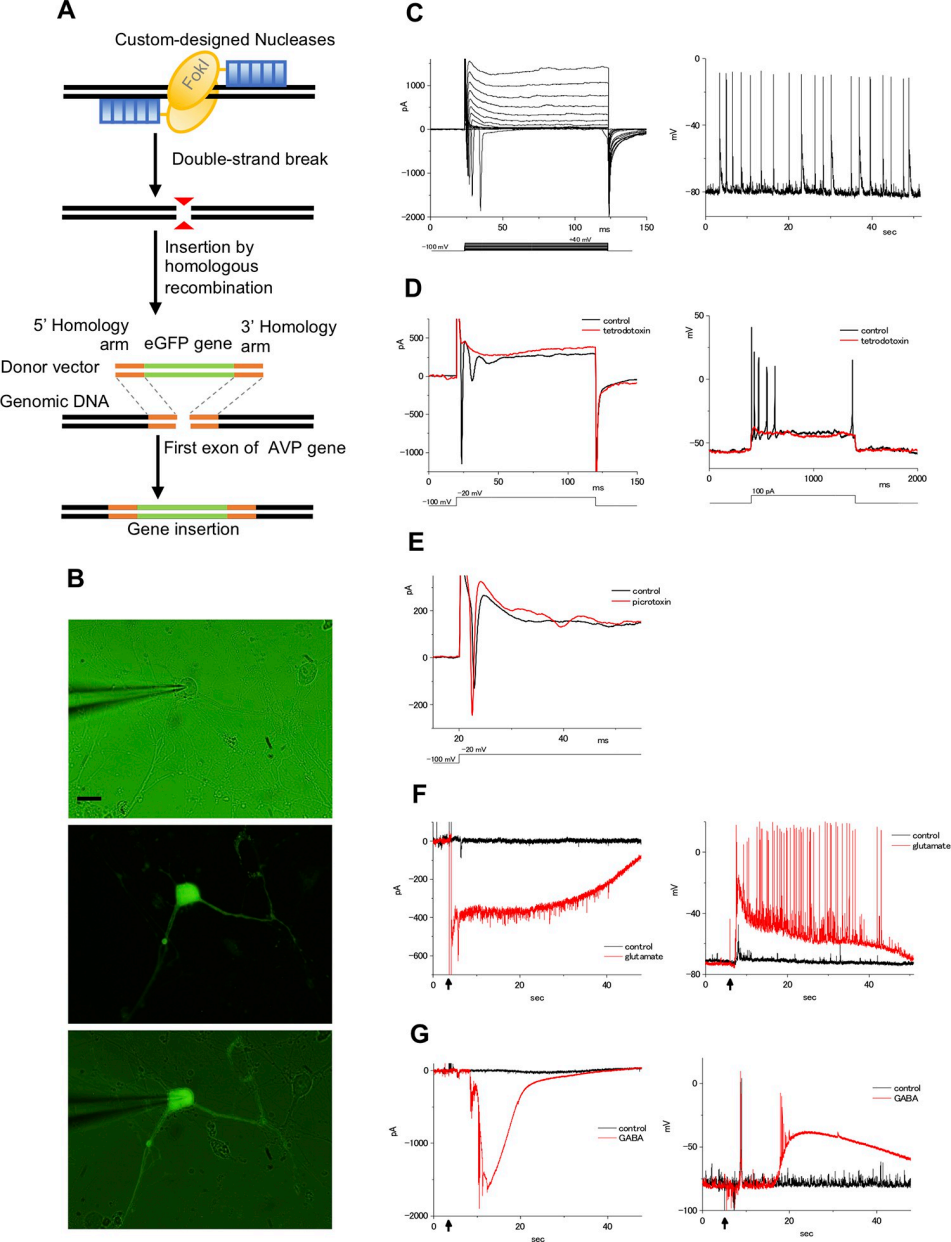
Electrophysiological recordings of AVP ^GFP/+^ cells. (A) Schematic illustration of TALEN-mediated knock-in of the GFP gene into the mAVP gene locus. (B-G) Whole-cell patch-clamp recordings of the AVP ^GFP/+^ culture cells. (B) Representative micrographs of the culture cells. The patch electrode approaches the soma of the cell from the left side (upper). Images show the GFP (middle) and Lucifer yellow fluorescence (lower) with transmitted light in the same cell as in the upper micrograph. Scale bars: 20 μm. (C) The membrane currents and spontaneous action potentials in the culture cells. Membrane currents in culture cells by depolarizing the voltage from a holding potential of –100 mV (left). Command voltages were increased in 10 mV steps from –100 mV to +40 mV. Spontaneous action potentials were observed in these cells under current-clamp conditions (right). No stimuli, such as current injections or neurotransmitters, were applied in this recording. (D) The inward current and action potential were blocked by tetrodotoxin (TTX). Membrane currents in culture cells by depolarizing the voltage from a holding potential of –100 mV (left). Command voltages were increased to −20 mV in control ringer solution (black line) or 1 μM TTX (red line). Responses to depolarization induced by injection of a 100 pA current, recorded in control ringer solution (control: black line), and the addition of 1 μM TTX (red line; right). (E) The effect of picrotoxin (PTX). Membrane currents in culture cells by depolarizing the voltage from –100 to −20 mV in control ringer solution (control: black line) or 10 μM PTX (red line). (F) The response to glutamate in the culture cells. Membrane currents were recorded under voltage-clamp conditions (left). Glutamate (up to 50 μM final concentration; red line) or ringer solution (control: black line) was applied at the arrow part. Membrane voltages were recorded under current-clamp conditions (right). (G) The response to GABA in the culture cells. Membrane currents were recorded under voltage-clamp conditions (left). GABA (up to 60 μM final concentration; red line) or ringer solution (control: black line) was applied at the arrow part. Membrane voltages were recorded under current-clamp conditions (right). Although GABA increased the membrane voltage, no action potential was generated except for immediately after the reaction (red line).

To investigate whether the mESC-derived hypothalamic neurons were electro-physiologically functional, we performed patch-clamp recordings. For these experiments, we used a mAVP::eGFP knock-in cell line (AVP ^GFP/+^ cell) in which the GFP gene had been heterozygously inserted into the mouse AVP gene locus using the TALEN system (Fig 2A). AVP ^GFP/+^ cells were induced into hypothalamic neurons, and then GFP-expressing AVP neurons were used for the patch-clamp recordings (Fig 2B). First, we investigated the spontaneous activities of GFP^+^ cells because AVP neurons express characteristic spontaneous phasic bursts composed of long bursts that last from several seconds to minutes followed by silence of a similar duration [23, 24]. We found that GFP^+^ cells displayed not bursts but robust spontaneous firing in the unstimulated states (Fig 2C). Application of TTX, a blocker of voltage-gated sodium channels, inhibited the inward currents and action potentials in GFP^+^ cells. (Fig 2D). Furthermore, the inward currents were slightly enhanced by PTX, an antagonist of GABA_A_ receptors (Fig 2E). Next, we examined the effects of neurotransmitters on GFP^+^ cells. Applying Glu induced robust inward currents and evoked action potentials (Fig 2F). We also found that applying GABA induced inward currents and increased the membrane voltage, but these were transient events, and action potentials were not generated except for immediately after the reaction (Fig 2G). These results suggest that mESC-derived hypothalamic neurons are electrophysiologically functional neurons.

### The survival and axonal projections of transplanted mESC-derived hypothalamic neurons in host adult mouse brain

**Fig 3:**
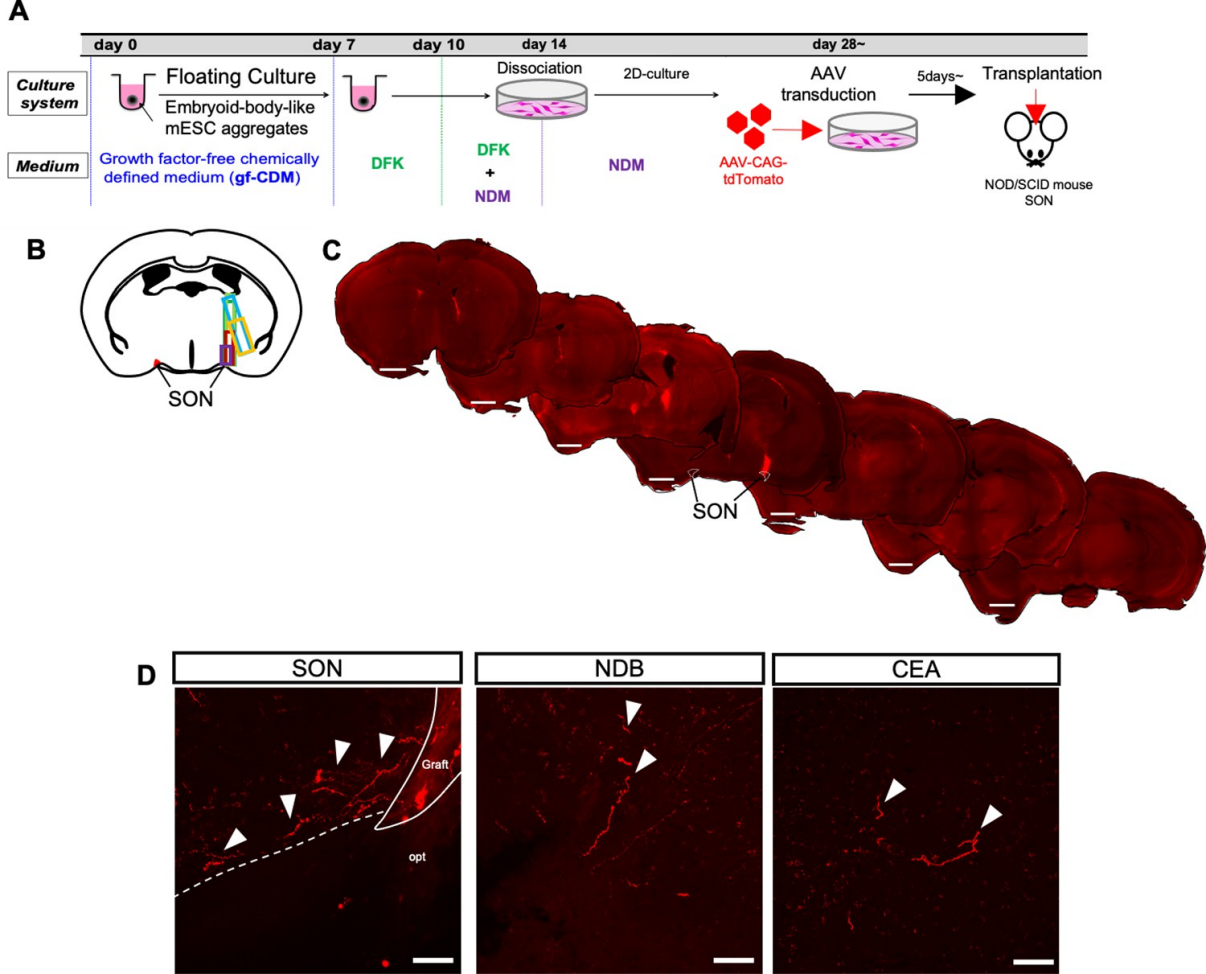
The survival and axonal projections of transplanted mESC-derived hypothalamic neurons into the SON at 3 months after transplantation. (A) Schematic illustration of the differentiation and transplantation protocol of mESCs. (B) The map of the graft location. Each graft is shown in different-colored squares. (C) Representative overviews of grafted mESC-derived hypothalamic neurons at 3 months after transplantation. Scale bars: 1 mm. (D) Representative Z stack maximum projection images of tdTomato^+^ graft-derived fibers at 3 months after transplantation in the SON, NDB and CEA. opt, optic tract. White arrowheads indicate tdTomato^+^ fibers. Scale bars: 50 μm.

Next, we examined whether mESC-derived hypothalamic neurons could survive and extend their axons in host adult mouse brain. At 28 days after induction, the differentiated hypothalamic neurons were transduced with AAV-CAG-tdTomato and then transplanted into the SON region of adult NOD/SCID mice (Fig 3A). We confirmed that the grafts survived until 3 months after transplantation without tumorigenesis. The graft survival rate was 0.8%±0.2% (n = 6) (S1 Data). We found that most of the grafted cells were located around the SON after 3months (Fig 3B and 3C). In some cases, we also found grafted cells in the lateral ventricle or the third ventricle, perhaps due to leakage during the operation.

We then examined the axonal projections of the grafted cells at 3 months after transplantation. tdTomato^+^ fibers showed a wide distribution across various brain regions (S2 Table). To clarify the relationship between axonal projections and the grafted cells, we transplanted cells into the SNr, which is adjacent to the lateral hypothalamic area of the hypothalamus. SNr neurons are known to innervate mainly the thalamus and midbrain [25]. At 3 months after transplantation, we that confirmed SNr-grafted cells survived at almost the same rate (0.7%±0.2%, n = 5) as the SON-grafted group (p = 0.67) (S1 Data). Most cells were located in the SNr region, but some were also found in the lateral and third ventricle (S1A and S1B Fig). The axonal projections of the SNr-grafted group overlapped with those of the SON-grafted group in 25 of 67 regions (S2 Table). Among these brain regions, we examined the projection of graft-derived axons in regions where intrinsic SON and SNr neurons project axons. Projection sites for SON neurons include the diagonal band nucleus (NDB), nucleus accumbens (NAc) shell, lateral septum (LS), and CA1, a known projection region for SON-OXT neurons [26], and central amygdala nucleus (CEA), a known projection region for SON-AVP neurons [27]. In contrast, thalamus, superior colliculus, pedunculopontine tegmental nucleus (PPTg), periaqueductal gray matter (PAG) and deep mesencephalic nucleus (DpMe) are known projection sites of intrinsic SNr neurons [25]. All of these SON- or SNr-specific projection regions showed graft projection in S2 Table, with the exception of PPTg, which was not examined in this study due to a lack of brain sections.

Because the above regions included areas in the vicinity of the ventricles, where the involvement of grafts in the ventricles could not be ruled out, they were excluded, leaving NDB, CEA, and DpMe remaining. The projection of graft-derived axons in these brain regions was specific to either SON or SNr (Table 1). The SON-grafted group was specifically distributed in the NDB and CEA (Fig 3D and Table 1). In contrast, the SNr-grafted group projected into the DpMe (S1C Fig and Table 1). These results indicate that axonal projection of the graft depends on the site of implantation and that the graft may project axons into the same region where the recipient site originally projected.

**Table 1:** Distribution of tdTomato^+^ graft-derived fibers in specific target regions of SON or SNr neurons.

			SON-grafted		SNr-grafted	
			% of mice with tdTomato signals within the region	No. of mice with tdTomato signals within the region	% of mice with tdTomato signals within the region	No. of mice with tdTomato signals within the region
Brain	SON-specific region	NDB	75%	(6 of 8)	0%	(0 of 8)
		CEA	55.6%	(5 of 9)	0%	(0 of 8)
	SNr-specific region	DpMe	0%	(0 of 8)	50%	(4 of 8)
Pituitary gland	SON-specific region	PPit	50%	(4 of 8)	0%	(0 of 8)
		IL	0%	(0 of 8)	0%	(0 of 8)
		Apit	0%	(0 of 8)	0%	(0 of 8)

This table shows the percentage and number of mice in which tdTomato^+^ fibers were observed within each brain region. The percentages were calculated from the number of mice shown in the right column (e.g. for the NDB of SON-grafted group, the statement “6 of 8” means that the total number of mice analyzed was “8”, and “6” of those mice had tdTomato^+^ fibers in the NDB; the percentage was then calculated as follows: (6/8) × 100 = 75%).

### Transplanted mESC-derived hypothalamic neurons extend their axons along with host neurons

**Fig 4:**
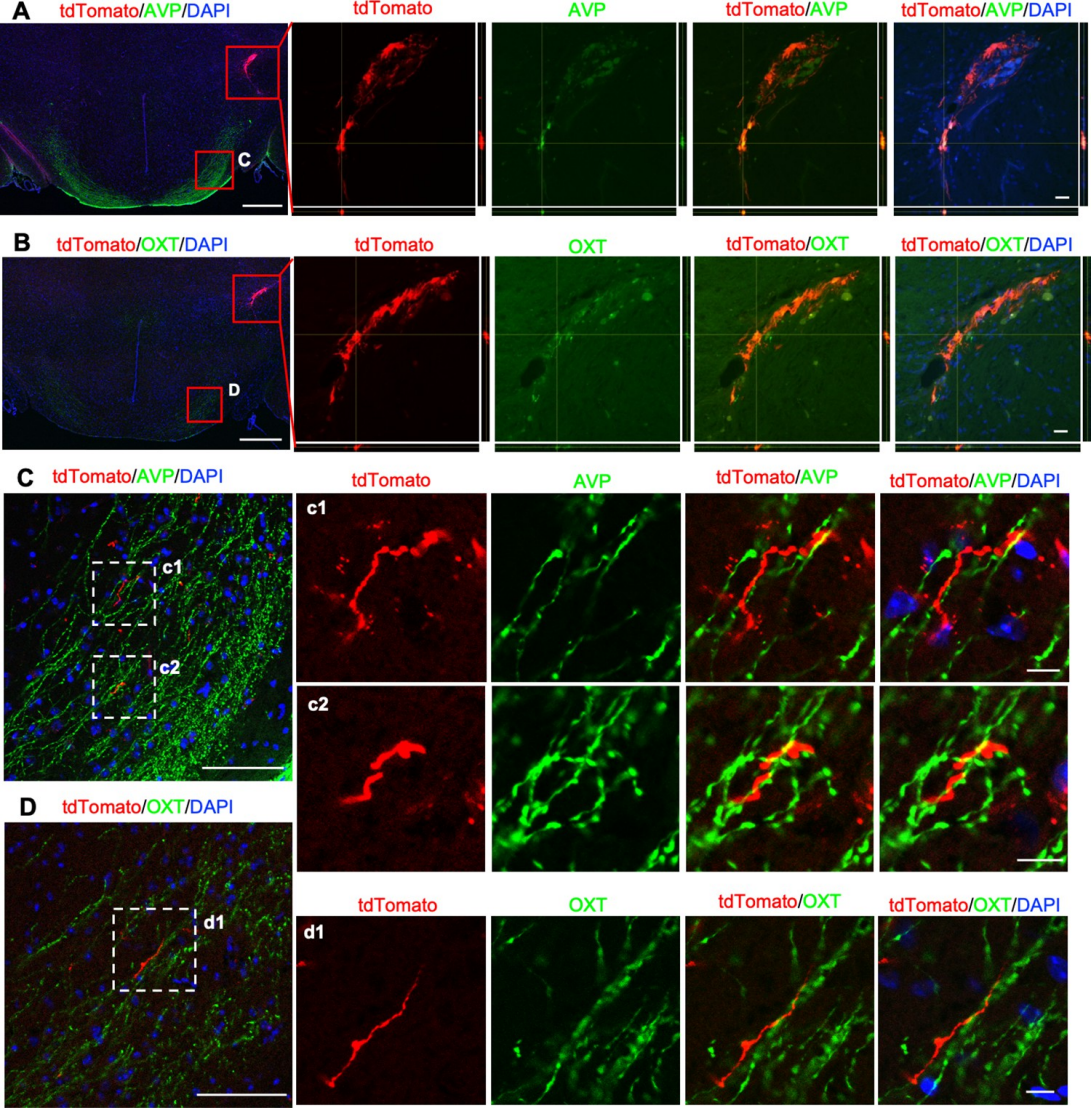
Immunohistochemistry for AVP or OXT on transplanted mESC-derived hypothalamic neurons at 3 months after transplantation. (A, B) Left panels show immunostainings for AVP (A) or OXT (B) in the hypothalamus at 3 months after transplantation. Right panels show enlarged images of grafts depicted in the upper red squares in left panels. Z stack maximum projection images and orthogonal views show weak AVP or OXT immunoreactivities colocalized with tdTomato signals. Bottom, x-z plane; right, y-z plane. Scale bars: 500 μm (left), 20 μm (right). (C, D) Left panels show enlarged images of the supra-opticohypophysial tract depicted in lower red squares in (A) or (B). (c1, 2) and (d1) show enlarged images depicted in white dotted line squares in left panels. Scale bars: 100 μm (left), 10 μm (right).

To investigate the graft-host interactions, we examined the distribution of tdTomato signals along with immunohistochemical signals for endogenous neuropeptides in the hypothalamus at 3 months after transplantation in the SON. We first checked the presence of the neuropeptides in the graft itself. Although host SON neurons abundantly express AVP and OXT, we observed not-neuron-like, weak AVP and OXT signals in the tdTomato^+^ grafts (Fig 4A and 4B). We also analyzed other neuropeptide markers, such as MCH, POMC and NPY. While the expression of MCH was similar to that of AVP and OXT (S2A Fig), the expression of POMC or NPY was hardly detectable in the grafts (S2B and S2C Fig).

Next, we focused on the ventral hypothalamic area apart from the graft site. We found that the tdTomato^+^ axons extended parallel to the host AVP^+^ and OXT^+^ supra-opticohypophysial tract in four out of eight in the SON-grafted group (Fig 4C and 4D). Similar observations were performed on the sections immunostained for MCH, POMC and NPY. MCH^+^ or POMC^+^ fibers were absent, and only tdTomato^+^ fibers were detected in the ventral hypothalamic area (S2D and S2E Fig). In contrast, both NPY^+^ fibers and tdTomato signals were detected in the ventral hypothalamic area, and the tdTomato signals existed in proximity to the NPY^+^ fibers (S2F Fig). NPY-containing neurons are known to project into the pituitary [28]. These results suggest that the graft-derived axons extend along with the host hypothalamic neuronal axons that project into the pituitary.

### Transplanted mESC-derived hypothalamic neurons project into the PPit

**Fig 5:**
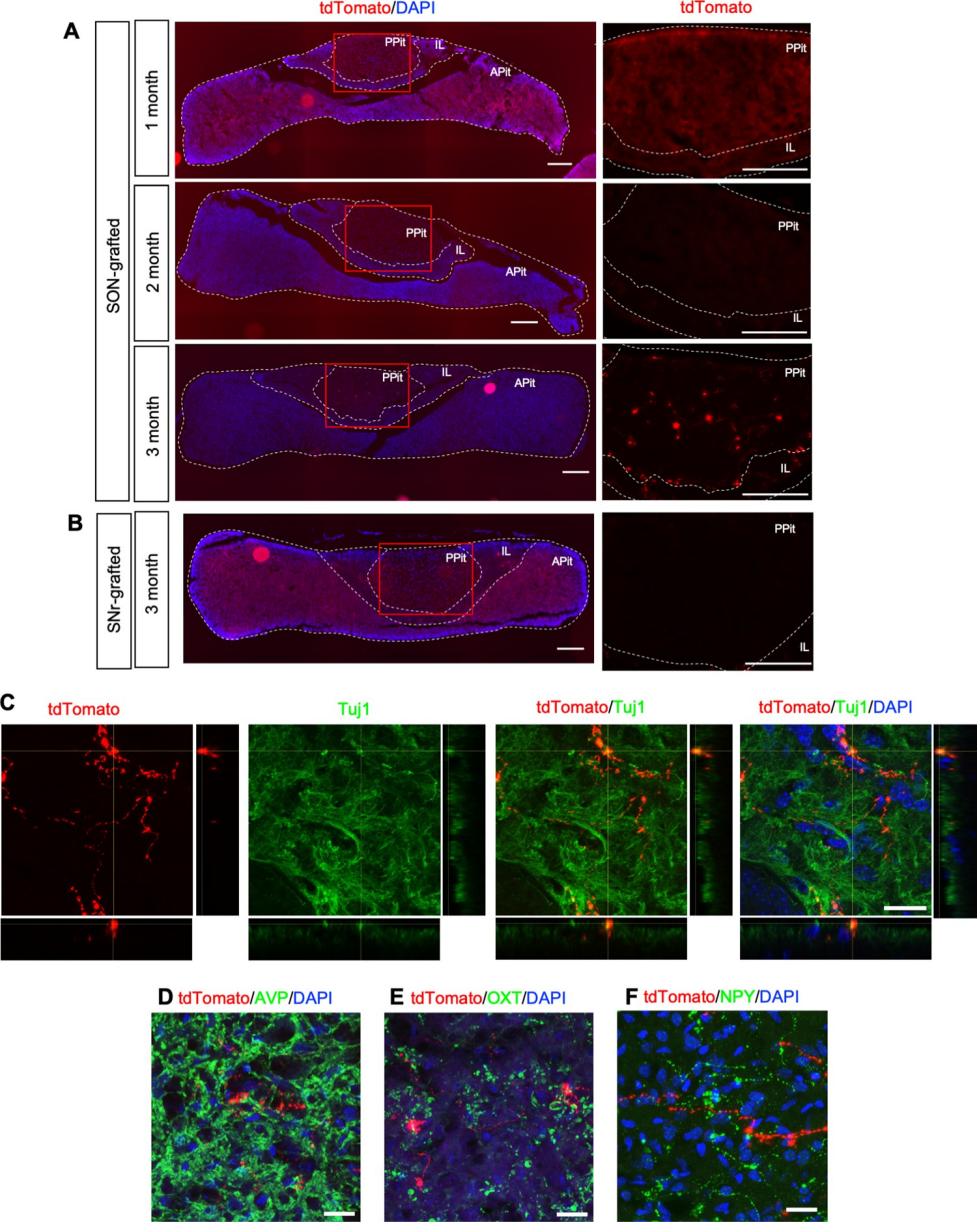
Graft-derived axons project into the PPit. Photographs of the pituitary at 1, 2 and 3 months after transplantation in the SON (A) or SNr (B). Left panels show the overviews of the pituitary. Right panels show enlarged images depicted in red squares in left panels. Scale bars: 200 μm. (C) Immunostaining for Tuj1 in the PPit at 3 months after transplantation. Z stack maximum projection images and orthogonal views demonstrate co-localization with the tdTomato signal and Tuj1 immunoreactivity. Bottom, x-z plane; right, y-z plane. Scale bars: 20 μm. (D, E, F) Z stack maximum projection images of the PPit immunostained for AVP (D), OXT (E) and NPY (F) at 3 months after transplantation. Scale bars: 20 μm.

Next, we examined the neural projections from the grafts into the pituitary. As a result, 50% of the grafts in the SON-grafted group showed intense tdTomato signals in the PPit but not in the intermediate lobe (IL) or anterior pituitary (APit) at 3 months after transplantation (Fig 5A and Table 1). tdTomato signals in the PPit were specific for SON and not detected in the SNr-grafted group at 3 months after transplantation (Fig 5B and Table 1). These results suggest the strong possibility that the tdTomato^+^ signals in PPit were axons extended from the orthotopically grafted cells in the SON. In addition, these tdTomato signals in the PPit were not detected at 1 or 2 months after transplantation (Fig 5A). As the distance between the graft region and the PPit was about 3.8 mm, it was speculated that the grafts extended their axons at a rate of 1.2–1.3 mm/month. To further confirm the graft-derived axon projections to the PPit, we transplanted cells into the SON, LPO or hippocampus of adult XSCID rats. As a result, only the grafts in the SON projected their axons into the PPit (S3 Fig). These results suggest that the graft-derived axon projections to the PPit are induced only when there is a match between the graft cell phenotype and the location.

To determine the identity of the tdTomato signals in the PPit, we conducted immunostaining for the neuronal markers Tuj1, AVP or OXT and captured Z stack images. The tdTomato signals partially overlapped with Tuj1 immunostaining (Fig 5C), suggesting that the tdTomato signals in the PPit were neuronal axons derived from the SON grafts. We found that the tdTomato signals ran though the AVP^+^ or OXT^+^ areas but did not overlap with AVP or OXT immunostaining (Figs 5D, 5E and S4, S5). In addition, we found that the tdTomato signals did not overlap with NPY staining (Figs 5F and S6). Taken together, these results suggest that the graft-derived tdTomato^+^ neuronal axons project from the SON to the PPit, but what type of neurons project into the PPit remains unclear.

## Discussion

In the present study, we performed orthotopic transplantation of mESC-derived hypothalamic neurons. We observed that the transplanted mESC-derived hypothalamic neurons survived for at least 3 months in host adult mouse brain. Furthermore, we found that the transplanted mESC-derived hypothalamic neurons showed long-range axonal projections into the target brain regions, including the PPit, corresponding to the host hypothalamic neurons.

In this study, we followed Wataya et al.’s SFEBq/gfCDM method [6] and confirmed that this method induced major types of hypothalamic neurons, as illustrated in previous studies [9, 11]. Electrophysiological studies showed that AVP neurons generated from AVP ^GFP/+^ cells using this method were electrically active. AVP neurons display a unique firing pattern characterized by a succession of periods of burst and silence, which is modified by the plasma osmolarity and various neuromodulators [29, 30]. In the AVP ^GFP/+^ cells, we confirmed spontaneous discharges, but the periodic bursts were not observed. It is believed that a neural circuit is required for the burst generation, as they are observed *in vivo* and in brain slices but not in isolated AVP neurons after enzymatic dispersion [31]. In our experiments, the differentiated AVP neurons on 2D-culture making contact with the surrounding cells displayed spontaneous discharges similar to those seen in isolated AVP neurons. Therefore, it is assumed that the hypothalamic cells derived from mESCs lack the crucial components required for burst generation.

Cell maturation is another crucial component for the electrophysiological properties of neurons. Rajamani et al. showed that the spontaneous activity of hiPSC-derived hypothalamic-like neurons (iHTNs) gradually increased from day 17 until day 50, suggesting that iHTNs began expressing the complement of ion channels and underwent the process of maturation during this period [32]. Yet another study showed that astrocyte co-culture enhanced the neuronal maturation and increased the maximal number of action potentials of human ES cell (hESC)-derived hypothalamic ARC-like neurons [33]. Based on the findings from these studies, the electrical properties of stem cell-derived hypothalamic neurons appear to be markedly affected by the degree of maturation and culture conditions, such as the culture period and existence of astrocytes.

In anticipation of regenerative applications, we transplanted mESC-derived hypothalamic neurons into the SON or SNr of NOD/SCID mice. Although we found that the grafts survived for at least 3 months after transplantation in the host brain, the survival rates of both the SON- (0.8%±0.2%; n = 6) and SNr-grafted group (0.7%±0.2%; n = 5) were relatively low. Consistent with these results, the low survival rate of grafted cells remains a major challenge to overcome. For example, in the case of stem cell transplantation in a Parkinson’s disease model, <300 tyrosine hydroxylase (TH)-positive neurons survived after transplanting 100,000–400,000 ES cells, and <4.3% of neural stem cell (NSC)-derived TH positive neurons survived after transplantation; these survival rates remain low even at present [34]. Reportedly around 90% of cells die during or in the first few days after transplantation by apoptosis [35]. In addition, a recent study demonstrated that a gradual increase in the number of apoptotic cells was observed in grafted hiPSC-derived substantia nigra pars compacta dopaminergic (SNpc DA) neurons from 1 to 12 months post-transplantation [36]. Whether or not a similar time course of apoptotic cell death occurred in our grafted mESC-hypothalamic neurons is unclear, so a further investigation will be required to improve the survival rate.

In the present study, we found grafted cells located in both the target region as well as the ventricles (Figs 3C and S1B). The survival of grafted cells in the ventricles made it difficult to analyze the precise axonal projections of grafted mESC-derived hypothalamic neurons, as the ventricles connected and enable the grafted cells to access a wide area of the brain. Therefore, axonal projections derived from transplanted cells were found in a wide area of the brain, making it impossible to determine whether they originated from grafts in target regions, such as the SON and SNr, or from grafted cells in the ventricles.

However, we observed some preferences with regard to axonal projections between the SON and SNr-grafted groups (Table 1, Figs 3D, S1C and S2 Table). For example, PPit, NDB and CEA are specific for the SON-grafted group, while DpMe is specific for the SNr-grafted group (Table 1). These results were consistent with the axonal projections of endogenous SON and SNr neurons [25–27]. While the precise mechanism underlying axonal projection remains unclear, we assume that the graft-derived innervation is affected by two factors: the location and cell phenotype. Regarding the graft location, it was demonstrated that mESC or hPSC-derived visual cortical cell-transplants successfully established an axonal pathway when they were grafted into the visual cortex but not the motor cortex [37, 38]. This suggests that the areal identity match is important for successful integration in the host brain. As for the cell phenotype, ventral midbrain (VM)-patterned hESC transplants displayed similar axonal projections and innervated appropriate targets for dopaminergic neuron, such as the dorsolateral striatum, NAc and ventromedial prefrontal cortex, when grafted either orthotopically or ectopically [39]. A recent study also demonstrated that two types of hESC-derived cells, midbrain dopamine (mDA) and Glu neurons, transplanted into the same location the SN of Parkinson’s disease mice projected axons to the different brain regions [40]. These findings suggest that graft innervation was determined principally by the cell phenotype. In the present study, we transplanted the same mESC-derived hypothalamic neurons into the SON and SNr and found that the two grafted groups showed different axonal projection patterns (Tables 1 and S2). In addition, we found that graft-derived axonal projections into the PPit were observed only when the grafts were located in the SON of both mice and rats but not when transplanted into the SNr of mice or LPO and the hippocampus of rats (Table 1, Figs 5A and 5B and S3). Our present results underscore the importance of areal identity, and we speculated that an areal identity-match was involved in the regulation of the axonal projections of grafted cells to the appropriate target regions. We did not examine the cell phenotype hypothesis in the present study. To that end, it would be worth comparing whether or not hypothalamic or non-hypothalamic neurons transplanted into the SON project their axons into the PPit.

**Fig 6:**
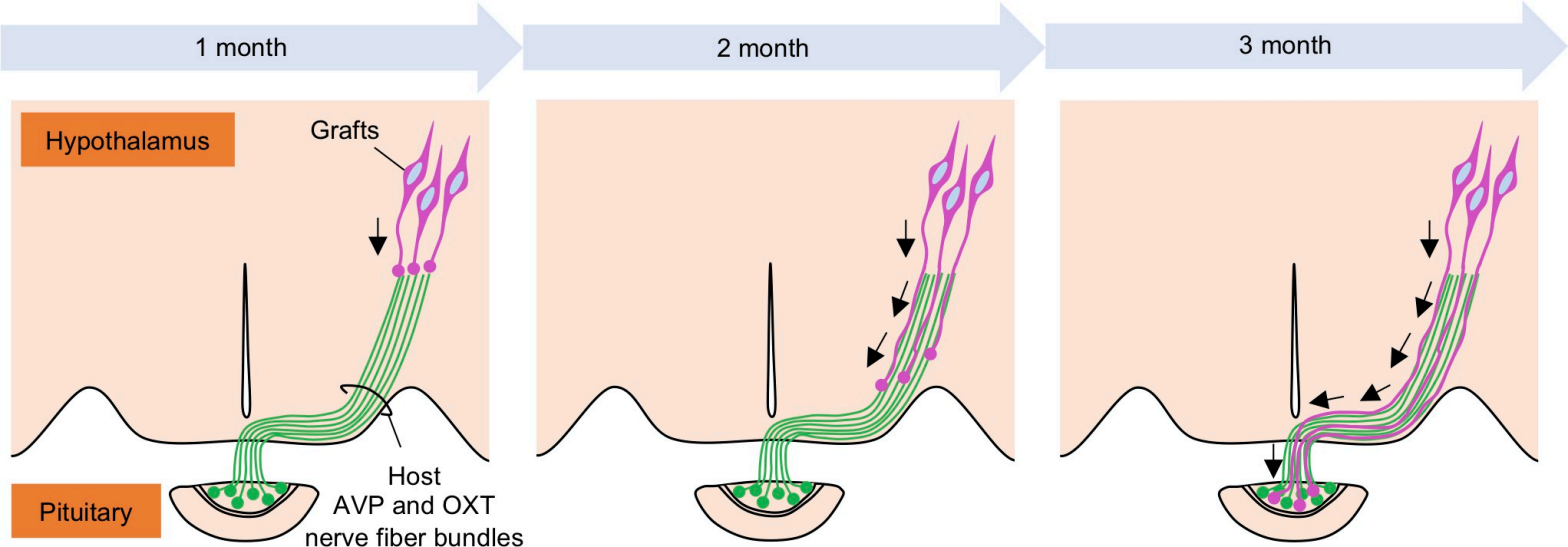
Possible mechanism for the axon elongation of grafts. A schematic illustration of the axonal elongation from grafts along the host AVP and OXT nerve fiber bundles. The nerve bundles may act as guidance cues for the grafts. It takes 3 months before the graft-derived axons reach the PPit.

One hypothetical explanation for the areal identity match is the guidance of graft-derived axons by the host nerve fiber bundles located close to the graft (Fig 6). Our data showed that the grafts in the SON elongated their axons alongside the host preexisting AVP or OXT axon bundles (Fig 4). The grafts in the SNr, LPO or hippocampus could not reach the PPit, probably because these areas do not have axon bundles projecting to the PPit. This notion is supported by a previous study that found that human ES cell-derived neurons grafted into the adult cortex or hippocampus developed region-specific efferent projections, and many of the newly formed axonal projections passed myelinated fiber tracts, such as the corpus callosum and fimbria-fornix [41]. Interestingly, a similar phenomenon occurs in the developing cerebral cortex. A previous study showed that immature neurites of multipolar cells make close contact with preexisting axons via the cell adhesion molecule transient axonal glycoprotein-1 (TAG-1), and then the immature neurites differentiate into axons and elongate along the preexisting axons [42]. Thus, we hypothesized that the axon elongation of grafts depends on the presence of host nerve fibers in the graft region. However, whether or not such cell adhesion molecules regulated the axon elongation of grafts in the present study remains unclear, so further studies should be conducted to clarify the precise molecular mechanism underlying the target projection of grafts.

Although our hypothalamic graft displayed similar axonal projection patterns to host hypothalamic neurons, we were unable to clarify which neurotransmitters they carried. We tested possible markers, including AVP, OXT, MCH, POMC and NPY, but none of them overlapped with the tdTomato signals. Further analyses will therefore be required to confirm this point.

In conclusion, our findings showed that functional mESC-derived hypothalamic neurons distributed their axons in the brain and PPit along with endogenous hypothalamic neurons when transplanted orthotopically. These results suggest the potential utility of stem cell-derived hypothalamic neurons in regenerative medicine for hypothalamus and pituitary diseases.

## Supporting information

S1 Data The minimal data set for the graft survival rate. Shown here is the graft volume, surviving cell number (before and after correction by the Abercrombie method), grafted cell number, % survival rate, mean, SD, SEM and statistical method and P value. 6 mice from the SON-grafted group and 5 from the SNr-grafted group were analyzed.
(XLSX)

S1 Fig The survival and axonal projections of transplanted mESC-derived hypothalamic neurons into the SNr at 3 months after transplantation. (A) The map of the graft location. Each graft is shown in different-colored squares. (B) Representative overviews of grafted mESC-derived hypothalamic neurons at 3 months after transplantation. Scale bars: 1 mm. (C) Representative Z stack maximum projection images of tdTomato^+^ graft-derived fibers at 3 months after transplantation in the SNr, the deep mesencephalic nucleus (DpMe). White arrowheads indicate tdTomato^+^ fibers. Scale bars: 50 μm.
(TIFF)

S2 Fig Immunohistochemistry for MCH, POMC or NPY on transplanted mESC-derived hypothalamic neurons at 3 months after transplantation. (A-C) Left panels show immunostainings for MCH (A), POMC (B) or NPY (C) in hypothalamus at 3 months after transplantation. Right panels show enlarged images of grafts depicted in upper red squares in left panels. White arrowheads indicate weak MCH immunoreactivities colocalized with tdTomato signals. Scale bars: 500 μm (left), 100 μm (right). (D-F) Each panel shows enlarged images of the ventral hypothalamic area depicted in lower red squares in (A-C). White arrowheads indicate tdTomato^+^ fibers. Scale bars: 100 μm.
(TIFF)

S3 Fig The comparison of the distribution of tdTomato signals in the pituitaries following transplantation into SON, LPO or hippocampus. (A-C) Left panels show representative coronal sections of pituitaries of grafts located in SON (A), LPO (B) and hippocampus (C) at 4 months, 3 months and 5 months after transplantation, respectively. Right panels show enlarged images depicted in red squares in left panels. tdTomato signals were detected in the pituitary of grafts located in the SON but not in the LPO or hippocampus. Scale bars: 200 μm. The protocol is described in S1 File.
(TIFF)

S4 Fig Immunohistochemistry for AVP in the pituitary at 3 months after transplantation. Single Z-plane images of the PPit immunostained with AVP as shown in Fig 5D. tdTomato signals were detected in the AVP immunoreactive areas but showed no notable overlap with AVP immunoreactivities. Scale bars: 20 μm.
(TIFF)

S5 Fig Immunohistochemistry for OXT in the pituitary at 3 months after transplantation. Single Z-plane images of the PPit immunostained with OXT as shown in Fig 5E. tdTomato signals were detected in the OXT immunoreactive areas but showed no notable overlap with OXT immunoreactivities. Scale bars: 20 μm.
(TIFF)

S6 Fig Immunohistochemistry for NPY in the pituitary at 3 months after transplantation. Single Z-plane images of the PPit immunostained with NPY as shown in Fig 5F. tdTomato signals and NPY immunoreactivities were in close proximity but showed no notable overlap. Scale bars: 20 μm.
(TIFF)

S1 Table List of primary and secondary antibodies used for immunocytochemistry and immunohistochemistry. (XLSX)

S2 Table Distribution and estimated density of tdTomato^+^ graft-derived fibers in host adult mouse brain and pituitary. The distribution and estimated density of tdTomato^+^ graft-derived fibers from the SON or SNr are shown here. The tdTomato^+^ fiber density was estimated from the mean percentage of the tdTomato^+^ area (μm^2^) in each brain region and classified as follows: high (+++), medium (++), low (+) and no fiber (-). The middle columns show the percentage of mice in which tdTomato^+^ fibers were observed within each brain region. These values were calculated from the number of mice shown in the right column. The right columns show the number of mice in which tdTomato^+^ fibers were observed within each brain region and the total number of mice analyzed in this study. For example, for the isocortex of the SON-grafted group, the statement “4 of 9” means that the total number of mice analyzed was “9”, and “4” of those mice had tdTomato^+^ fibers in the isocortex. The percentage was calculated as follows: (4/9) × 100 = 44.4%.
(PDF)

S1 File Transplantation of mESCs derived-hypothalamic neurons into the rat brain. (DOCX)
