# Supplementary figures and images for: Long-range axonal projections of transplanted mouse embryonic stem cell-derived hypothalamic neurons into adult mouse brain
Source: PLoS One. 2022 Nov 10;17(11):e0276694. doi: 10.1371/journal.pone.0276694 (PMC9648832; doi:10.1371/journal.pone.0276694)

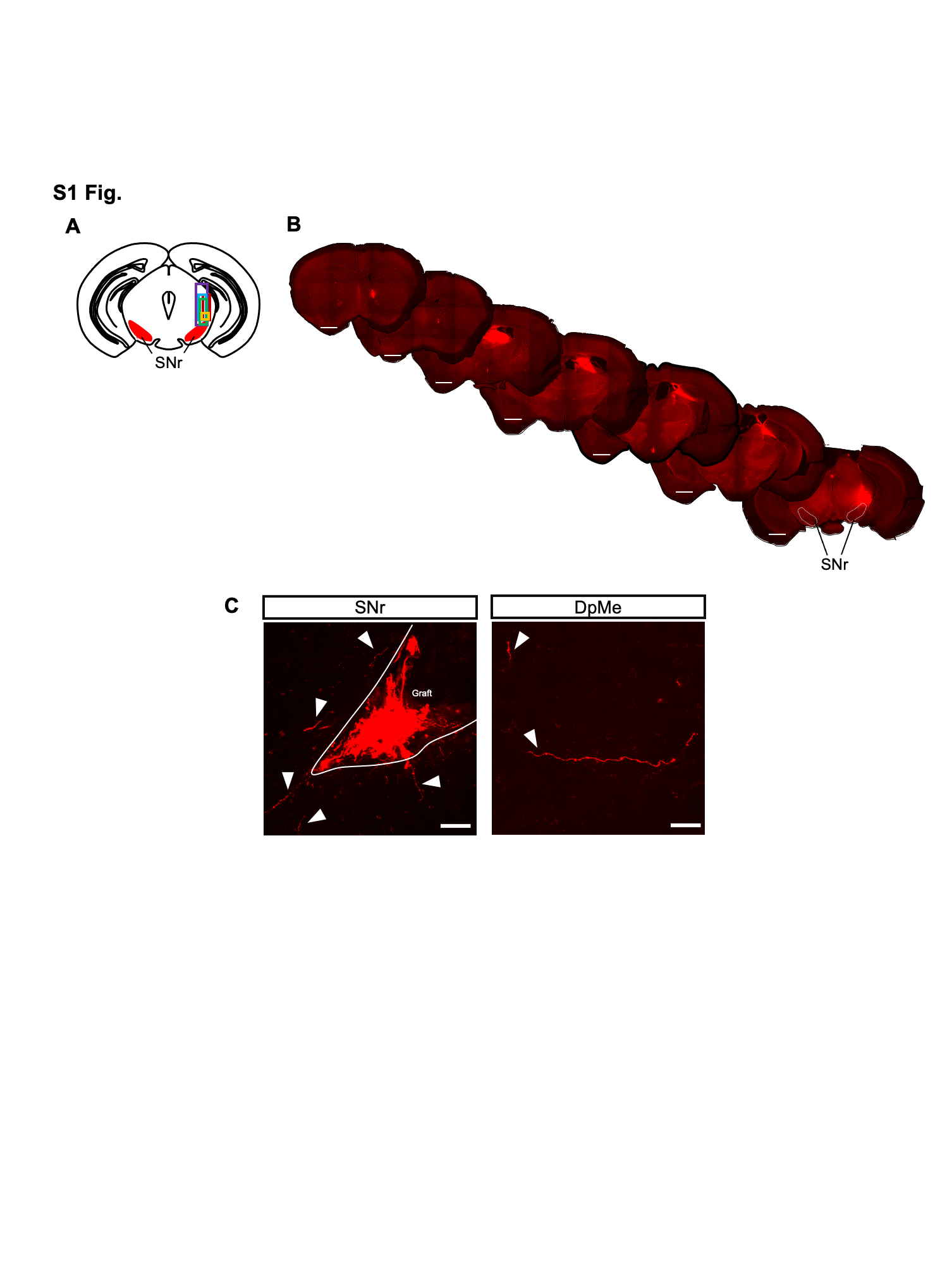

Supplement: S1 Fig — (A) The map of the graft location. Each graft is shown in different-colored squares. (B) Representative overviews of grafted mESC-derived hypothalamic neurons at 3 months after transplantation. Scale bars: 1 mm. (C) Representative Z stack maximum projection images of tdTomato+ graft-derived fibers at 3 months after transplantation in the SNr, the deep mesencephalic nucleus (DpMe). White arrowheads indicate tdTomato+ fibers. Scale bars: 50 μm. (TIFF) [file pone.0276694.s002.tiff]

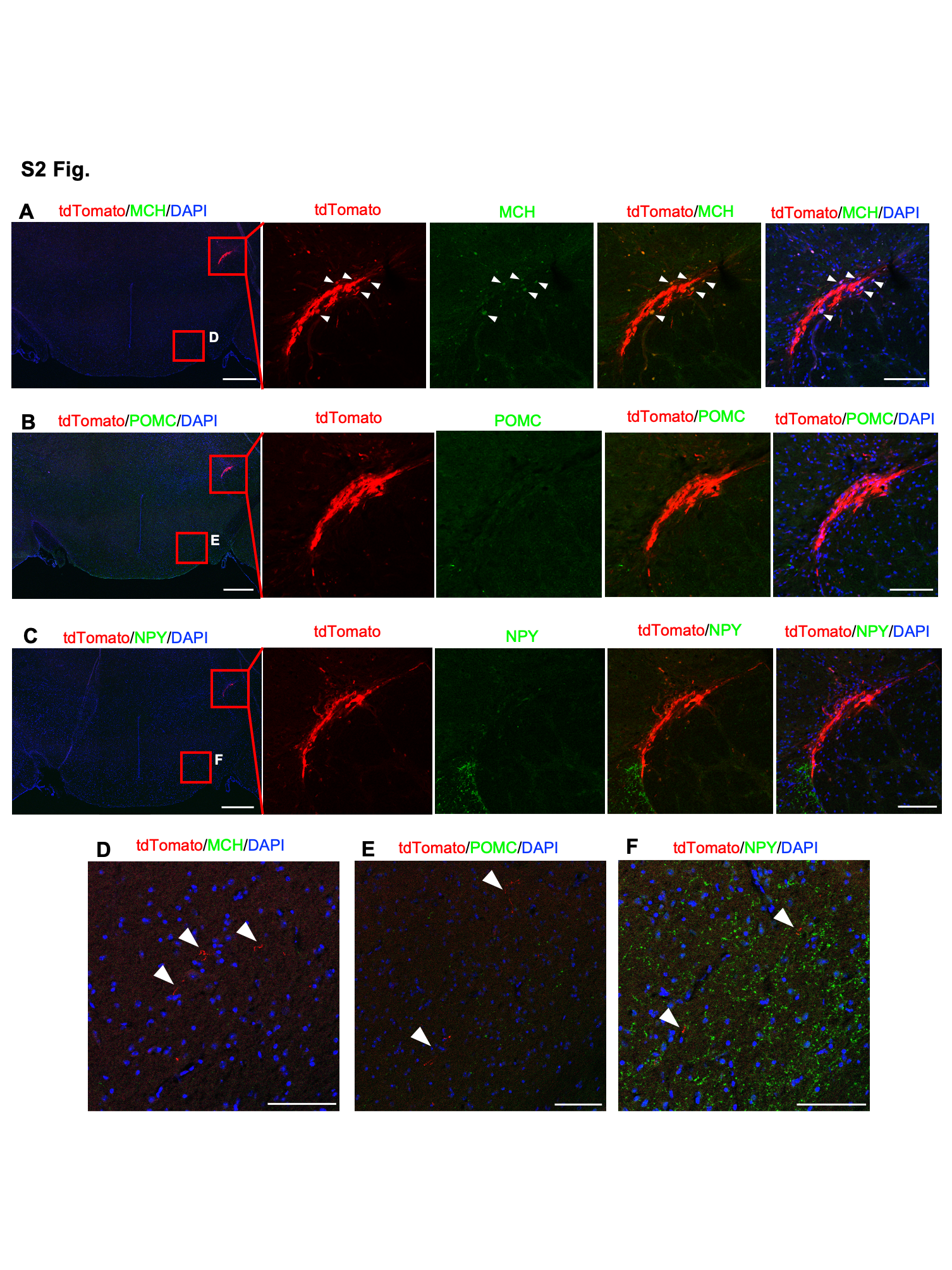

Supplement: S2 Fig — (A-C) Left panels show immunostainings for MCH (A), POMC (B) or NPY (C) in hypothalamus at 3 months after transplantation. Right panels show enlarged images of grafts depicted in upper red squares in left panels. White arrowheads indicate weak MCH immunoreactivities colocalized with tdTomato signals. Scale bars: 500 μm (left), 100 μm (right). (D-F) Each panel shows enlarged images of the ventral hypothalamic area depicted in lower red squares in (A-C). White arrowheads indicate tdTomato+ fibers. Scale bars: 100 μm. (TIFF) [file pone.0276694.s003.tiff]

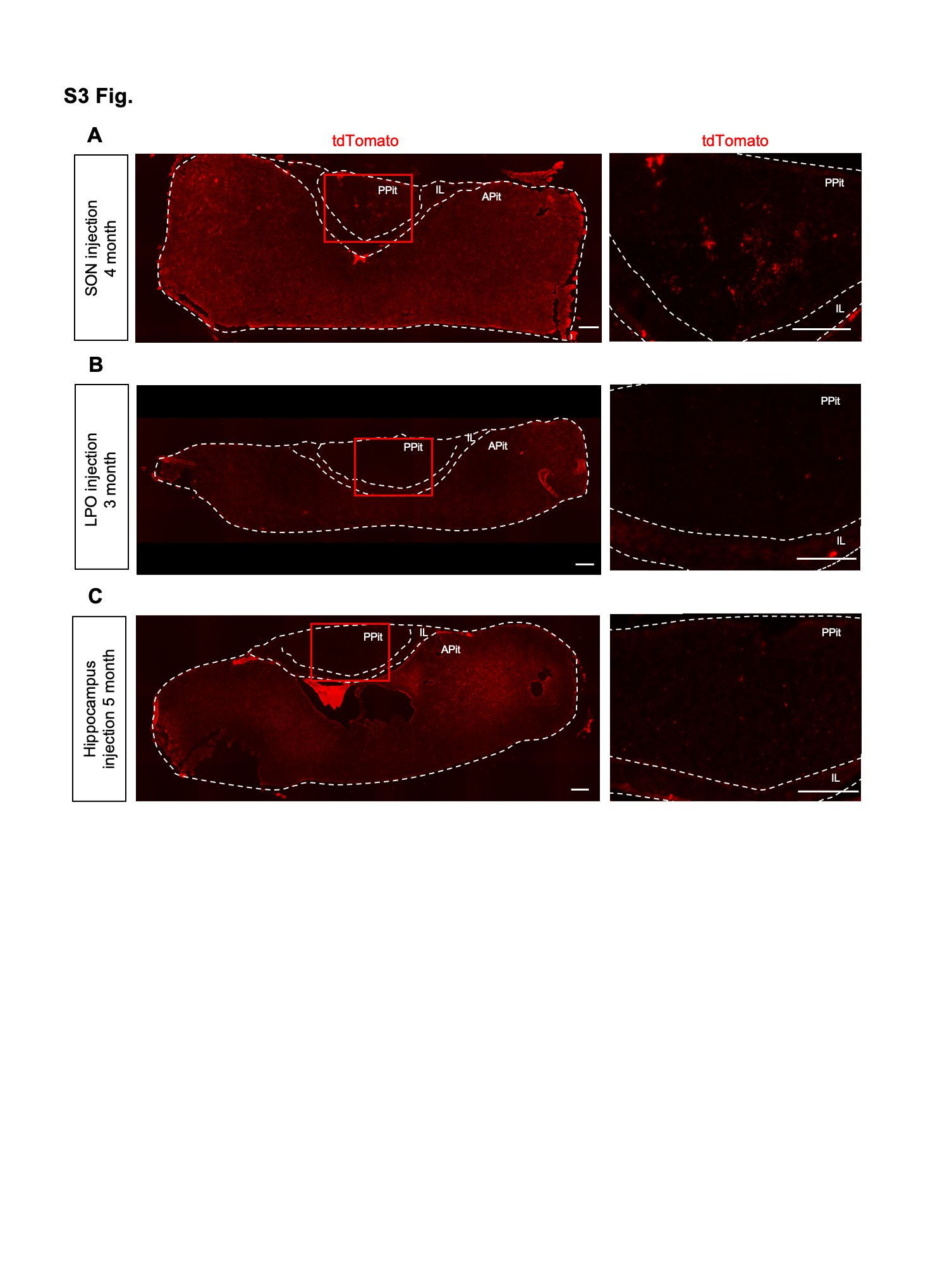

Supplement: S3 Fig — (A-C) Left panels show representative coronal sections of pituitaries of grafts located in SON (A), LPO (B) and hippocampus (C) at 4 months, 3 months and 5 months after transplantation, respectively. Right panels show enlarged images depicted in red squares in left panels. tdTomato signals were detected in the pituitary of grafts located in the SON but not in the LPO or hippocampus. Scale bars: 200 μm. The protocol is described in S1 File. (TIFF) [file pone.0276694.s004.tiff]

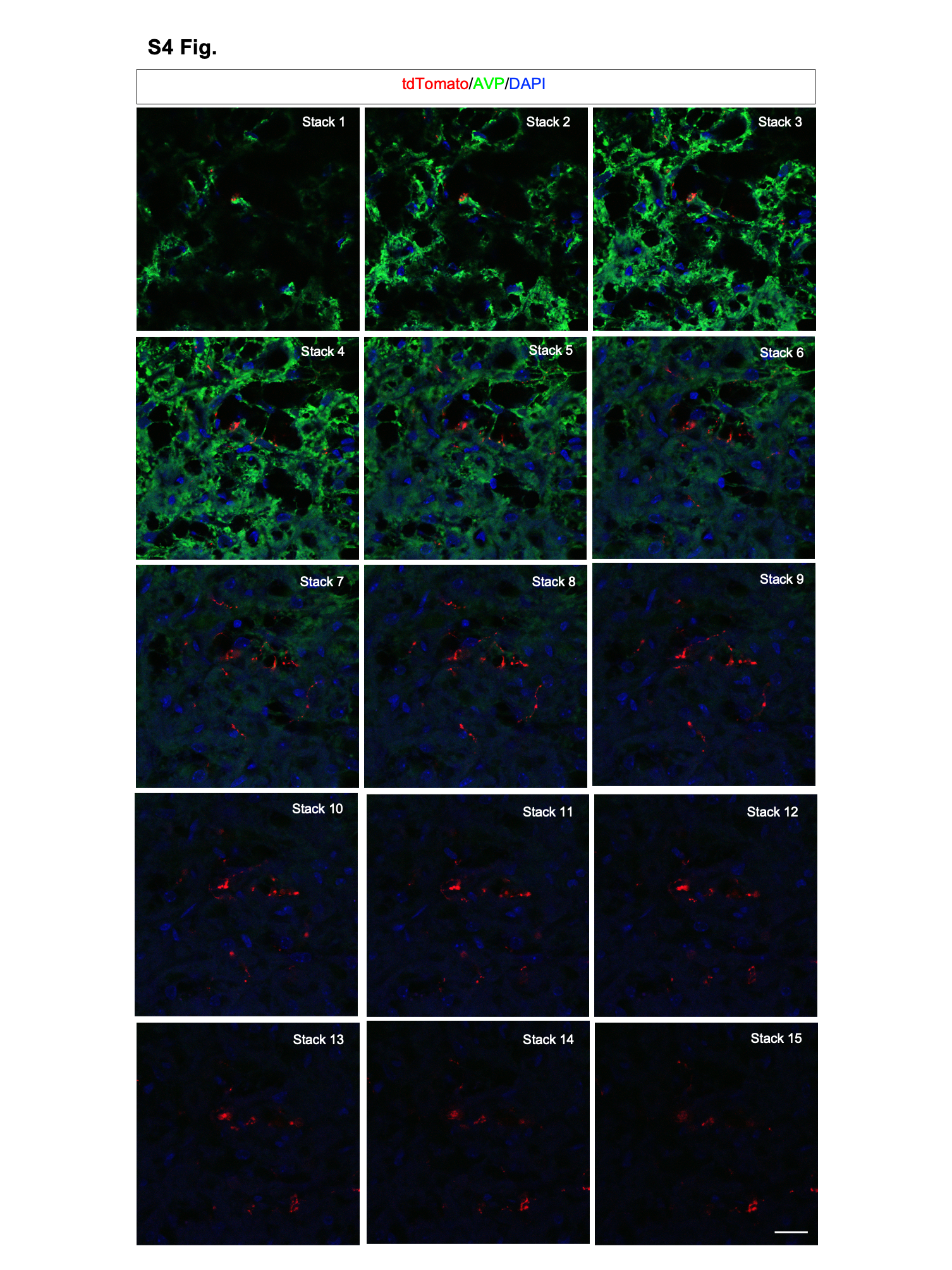

Supplement: S4 Fig — Single Z-plane images of the PPit immunostained with AVP as shown in Fig 5D. tdTomato signals were detected in the AVP immunoreactive areas but showed no notable overlap with AVP immunoreactivities. Scale bars: 20 μm. (TIFF) [file pone.0276694.s005.tiff]

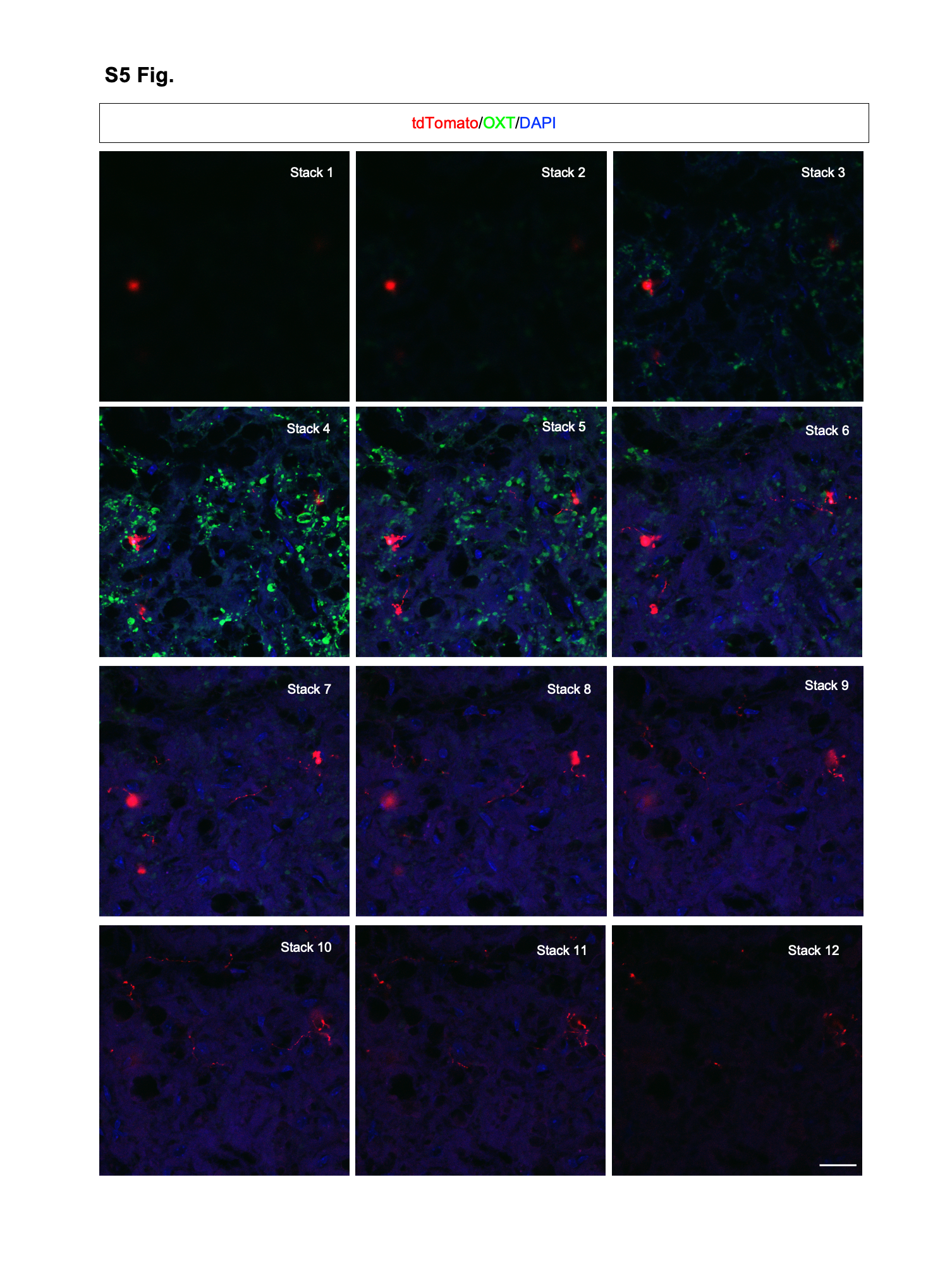

Supplement: S5 Fig — Single Z-plane images of the PPit immunostained with OXT as shown in Fig 5E. tdTomato signals were detected in the OXT immunoreactive areas but showed no notable overlap with OXT immunoreactivities. Scale bars: 20 μm. (TIFF) [file pone.0276694.s006.tiff]

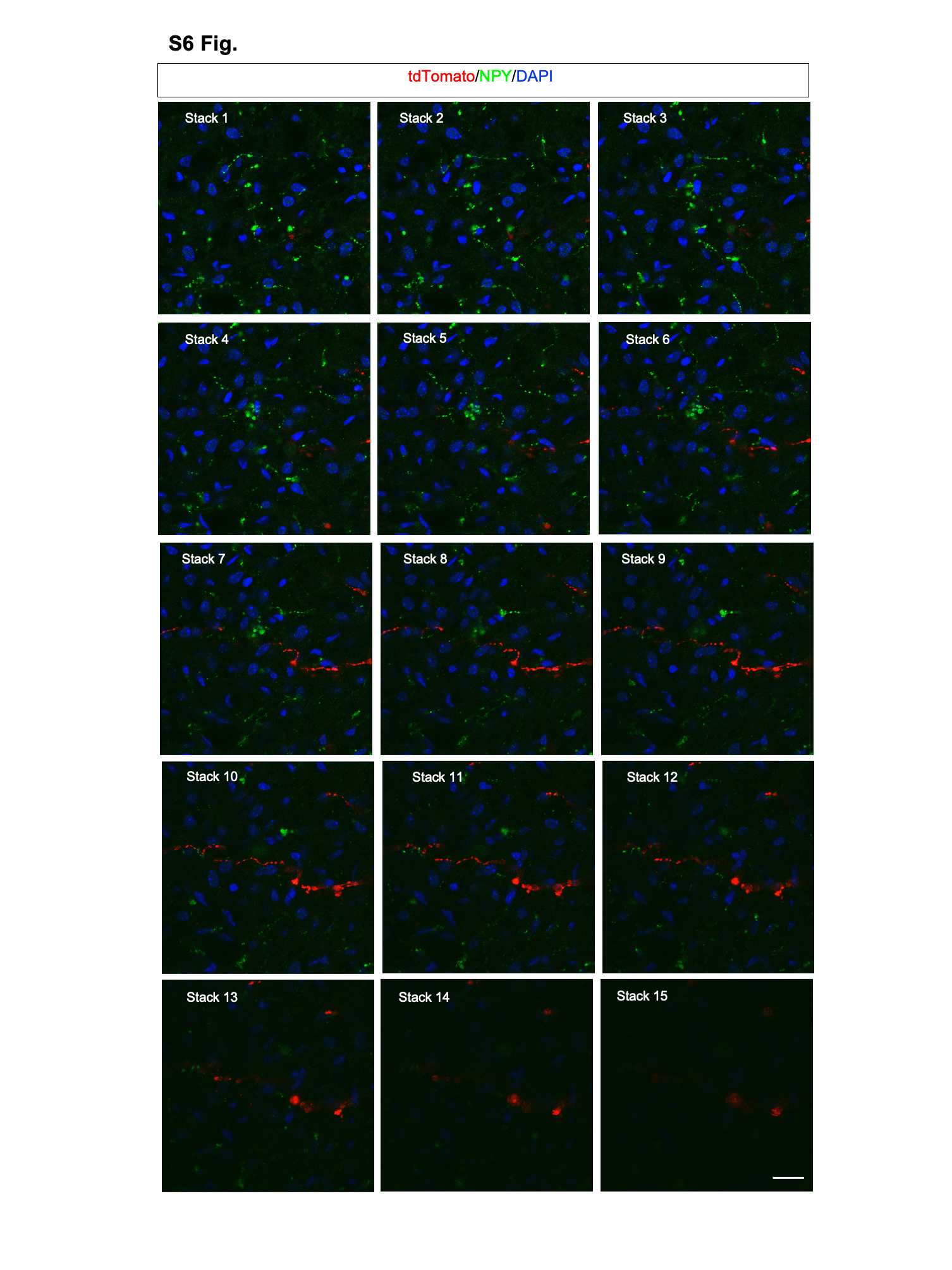

Supplement: S6 Fig — Single Z-plane images of the PPit immunostained with NPY as shown in Fig 5F. tdTomato signals and NPY immunoreactivities were in close proximity but showed no notable overlap. Scale bars: 20 μm. (TIFF) [file pone.0276694.s007.tiff]
