# Supplementary material for: Long-range axonal projections of transplanted mouse embryonic stem cell-derived hypothalamic neurons into adult mouse brain
Source: PLoS One. 2022 Nov 10;17(11):e0276694. doi: 10.1371/journal.pone.0276694 (PMC9648832; doi:10.1371/journal.pone.0276694)
Supplement: S2 Table — The distribution and estimated density of tdTomato+ graft-derived fibers from the SON or SNr are shown here. The tdTomato+ fiber density was estimated from the mean percentage of the tdTomato+ area (μm2) in each brain region and classified as follows: high (+++), medium (++), low (+) and no fiber (-). The middle columns show the percentage of mice in which tdTomato+ fibers were observed within each brain region. These values were calculated from the number of mice shown in the right column. The right columns show the number of mice in which tdTomato+ fibers were observed within each brain region and the total number of mice analyzed in this study. For example, for the isocortex of the SON-grafted group, the statement “4 of 9” means that the total number of mice analyzed was “9”, and “4” of those mice had tdTomato+ fibers in the isocortex. The percentage was calculated as follows: (4/9) × 100 = 44.4%. (PDF) [file pone.0276694.s009.pdf]

S2 Table. Distribution and estimated density of tdTomato+ graft-derived fibers in host adult mouse brain and pituitary.

	SON-grafted			SNr-grafted		
	Estimated fiber density	% of mice with tdTomato signals within the region	No. of mice with tdTomato signals within the region	Estimated fiber density	% of mice with tdTomato signals within the region	No. of mice with tdTomato signals within the region
**Brain region**						
**Cerebral cortex**						
Isocortex	+	44.4	4 of 9	+	25.0	2 of 8
Piriform area (PIR)	+	22.2	2 of 9	+	12.5	1 of 8
Cortical amygdalar area (COA)	+	66.7	6 of 9	-	0.0	0 of 8
**Striatum**						
Caudoputamen (CP)	+	77.8	7 of 9	+	12.5	1 of 8
Nucleus accumbens core (NAc core)	+	87.5	7 of 8	-	0.0	0 of 8
Nucleus accumbens shell (NAc shell)	+	100.0	8 of 8	+	12.5	1 of 8
Olfactory tubercle (OT)	+	50.0	4 of 8	-	0.0	0 of 8
**Septum**						
Lateral septal nucleus (LS)	+	87.5	7 of 8	+	12.5	1 of 8
Medial septal nucleus (MS)	+	25.0	2 of 8	-	0.0	0 of 8
Diagonal band nucleus (NDB)	+	75.0	6 of 8	-	0.0	0 of 8
**Pallidum**						
Bed nuclei of the stria terminalis (BST)	+	88.9	8 of 9	+	12.5	1 of 8
Globus pallidus	-	0.0	0 of 9	+	12.5	1 of 8
Substantia innominata (SI)	+	44.4	4 of 9	+	12.5	1 of 8
**Hypothalamus**						
Medial preoptic area (MPO)	+	66.7	6 of 9	+	12.5	1 of 8
Lateral preoptic area (LPO)	+	22.2	2 of 9	-	0.0	0 of 8
Suprachiasmatic nucleus (SCH)	-	0.0	0 of 9	+	12.5	1 of 8
Zona incerta (ZI)	+	22.2	2 of 9	+	25.0	2 of 8
Anterior hypothalamic nucleus (AH)	+	44.4	4 of 9	+	12.5	1 of 8
Paraventricular hypothalamic nucleus (PVH)	+	22.2	2 of 9	+	12.5	1 of 8
Supraoptic nucleus (SO)	+	66.7	6 of 9	-	0.0	0 of 8
Tuberal nucleus (TU)	+	44.4	4 of 9	-	0.0	0 of 8
Subthalamic nucleus (STN)	+	11.1	1 of 9	+	37.5	3 of 8
Lateral hypothalamic area (LHA)	+	77.8	7 of 9	+	37.5	3 of 8
Posterior hypothalamic nucleus (PH)	+	22.2	2 of 9	-	0.0	0 of 8
Dorsomedial nucleus of the hypothalamus (DMH)	+	33.3	3 of 9	-	0.0	0 of 8
Ventromedial hypothalamic nucleus (VMH)	+	44.4	4 of 9	-	0.0	0 of 8
Arcuate hypothalamic nucleus (ARH)	+	44.4	4 of 9	+	12.5	1 of 8
Mammilarly body (MBO)	+	25.0	2 of 8	-	0.0	0 of 8
**Thalamus**						
Medial habenula (MH)	+	11.1	1 of 9	-	0.0	0 of 8
Lateral habenula (LH)	-	0.0	0 of 9	+	12.5	1 of 8
Paraventricular nucleus of the thalamus (PVT)	-	0.0	0 of 9	-	0.0	0 of 8
Medial geniculate complex (MG)	-	0.0	0 of 8	+	12.5	1 of 8
Lateral group of the dorsal thalamus (LAT)	-	0.0	0 of 9	+	25.0	2 of 8
Anterior group of the dorsal thalamus (ATN)	+	22.2	2 of 9	-	0.0	0 of 8
Ventral group of the dorsal thalamus (VENT)	+	33.3	3 of 9	+	25.0	2 of 8
Subparafascicular nucleus (SPF)	+	11.1	1 of 9	-	0.0	0 of 8
Reticular nucleus of the thalamus (Rt)	+	22.2	2 of 9	-	0.0	0 of 8
**Hippocampus**						
CA1	+	44.4	4 of 9	+++	37.5	3 of 8
CA2	+	11.1	1 of 9	+	12.5	1 of 8
CA3	+	77.8	7 of 9	+	25.0	2 of 8
Dentate gyrus (DG)	-	0.0	0 of 9	+	37.5	3 of 8
**Amygdala**						
Lateral amygdalar nucleus (LA)	+	22.2	2 of 9	-	0.0	0 of 8
Basolateral amygdalar nucleus (BLA)	+	11.1	1 of 9	-	0.0	0 of 8
Basomedial amygdalar nucleus (BMA)	+	11.1	1 of 9	-	0.0	0 of 8
Central amygdalar nucleus (CEA)	++	55.6	5 of 9	-	0.0	0 of 8
Medial amygdalar nucleus (MEA)	++	66.7	6 of 9	-	0.0	0 of 8
Posterior amygdalar nucleus (PA)	-	0.0	0 of 8	-	0.0	0 of 8
**Midbrain**						
Periaqueductal gray (PAG)	-	0.0	0 of 8	+	12.5	1 of 8
deep mesencephalic nucleus (DpMe)	-	0.0	0 of 8	+	50.0	4 of 8
Substantia compact (SNc)	-	0.0	0 of 8	+	12.5	1 of 8
Substantia nigra (SNr)	+	50.0	4 of 8	+++	25.0	2 of 8
Ventral tegmental area (VTA)	+	50.0	4 of 8	+	25.0	2 of 8
Superior colliculus	-	0.0	0 of 8	+	12.5	1 of 8
Anterior pretectal nucleus (APN)	+	11.1	1 of 9	+	12.5	1 of 8
Interpeduncular nucleus (IPN)	+	12.5	1 of 8	-	0.0	0 of 8
Rostral linear nucleus raphe (RL)	-	0.0	0 of 8	+	12.5	1 of 8
**fiber tracts**						
corpus callosum (CC)	+	77.8	7 of 9	-	0.0	0 of 8
fimbria (fi)	+	44.4	4 of 9	+	12.5	1 of 8
ventral hippocampal commissure (vhc)	+	11.1	1 of 9	-	0.0	0 of 8
alveus (alv)	+	22.2	2 of 9	+	12.5	1 of 8
columns of the fornix (fx)	+	11.1	1 of 9	-	0.0	0 of 8
medial lemniscus	-	0.0	0 of 9	+	12.5	1 of 8
Internal capsule (int)	+	22.2	2 of 9	+	50.0	4 of 8
cerebral peduncle (cpd)	+	11.1	1 of 9	+	50.0	4 of 8
**Pituitary gland**						
Posterior pituitary (PPit)	+	50.0	4 of 8	-	0.0	0 of 8
Intermediate lobe (IL)	-	0.0	0 of 8	-	0.0	0 of 8
Anterior pituitary (APit)	-	0.0	0 of 8	-	0.0	0 of 8

The distribution and estimated density of tdTomato+ graft-derived fibers from the SON or SNr are shown here. The tdTomato+ fiber density was estimated from the mean percentage of the tdTomato+ area (μm^2) in each brain region and classified as follows: high (+++), medium (++), low (+) and no fiber (-). The middle columns show the percentage of mice in which tdTomato+ fibers were observed within each brain region. These values were calculated from the number of mice shown in the right column. The right columns show the number of mice in which tdTomato+ fibers were observed within each brain region and the total number of mice analyzed in this study. For example, for the isocortex of the SON-grafted group, the statement "4 of 9" means that the total number of mice analyzed was "9", and "4" of those mice had tdTomato+ fibers in the isocortex. The percentage was calculated as follows: (4/9) × 100 = 44.4%.
