# Supplementary material for: Long-range axonal projections of transplanted mouse embryonic stem cell-derived hypothalamic neurons into adult mouse brain
Source: PLoS One. 2022 Nov 10;17(11):e0276694. doi: 10.1371/journal.pone.0276694 (PMC9648832; doi:10.1371/journal.pone.0276694)
Supplement: S1 File — (DOCX) [file pone.0276694.s010.docx]

**S1 protocol. Transplantation of mESCs derived-hypothalamic neurnos into the rat brain.** X-SCID rats (F344-*Il2rg^em1Iexas^*, NBRP-Rat No.0883, Kyoto University, Japan, RRID: RGD_13464263 [1]) were kept under a 12 h light/dark cycle with access to food and water *ad libitum*. At 9- or 18-weeks old X-SCID rats were anesthetized with isoflurane and placed in a stereotaxic apparatus (Narishige, Tokyo, Japan; Cat# SR-6N). mESC-derived hypothalamic neurons resuspended at a density of 4 × 10^5^ to 5 × 10^5^ cells per rat were transplanted into the SON (from bregma: A -1.2 mm, L -1.9 mm, V +10.9 mm), LPO (from bregma: A +0.5 mm, L -2.5 mm, V +7.5 mm) or hippocampus (from bregma: A -3.0 mm, L -2.0 mm, V +3.0 mm) using a 22 gauge Hamilton microsyringe. For the observation of axons, coronal sections of pituitaries were prepared. In brief, to alleviate suffering, rats were deeply anesthetized with isoflurane before perfusion fixation. Rats were transcardially perfused with PBS followed by 4% PFA, and then their brains were collected. The brains were postfixed overnight in 4% PFA and cryoprotected in sucrose solutions of increasing concentrations (10%, 20% and 30%) overnight at 4 °C. Pituitaries were coronally sectioned (30 μm thick) on a freezing microtome (CM1950; Leica Microsystems, RRID:SCR_018061) and mounted onto CREST-coated slides (Matsunami Glass; Cat# SCRE). Imaging data were acquired using an inverted fluorescence microscope DMI6000B (Leica Microsystems, Manheim, Germany, RRID:SCR_020216) or BIOREVO BZ-9000 (Keyence, Osaka, Japan, RRID:SCR_015486) and analyzed with the Fiji software (RRID:SCR_002285).

**Supporting reference**

1. Mashimo T, Takizawa A, Voigt B, Yoshimi K, Hiai H, Kuramoto T, et al. Generation of knockout rats with X-linked severe combined immunodeficiency (X-SCID) using zinc-finger nucleases. PLoS One. 2010;5: e8870. doi:10.1371/journal.pone.0008870
